# A case report of iliopsoas abscess and literature review

**DOI:** 10.1097/MD.0000000000039356

**Published:** 2024-08-16

**Authors:** Wenyu He, Ye Yuan, Jihua Huang

**Affiliations:** a Intensive Care Unit, Guangdong Provincial People’s Hospital, Zhuhai Hospital (Jinwan Central Hospital of Zhuhai), Zhuhai, China; b Department of Nephrology, Guangdong Provincial People’s Hospital, Zhuhai Hospital (Jinwan Central Hospital of Zhuhai), Zhuhai, China.

**Keywords:** antibiotics, iliopsoas abscess, methicillin-sensitive *Staphylococcus aureus*, sepsis

## Abstract

**Rationale:**

Iliopsoas abscess is a rare acute medical condition. It usually occurs because of the spread of infection from adjacent structures and hematogenous spread. Clinical features include fever, backache, radiating nerve root pain, and leg weakness. When sepsis occurs, prompt recognition is required to initiate appropriate antimicrobial therapy and surgical drainage.

**Patient concerns:**

A 65-year-old male presented to the outpatient department with a 2-day history of lower back, hip, and leg pain, for which analgesics were administered. During hospitalization, he experienced deterioration, becoming febrile, hypoxic, hypotensive, tachycardiac, and delirious.

**Interventions:**

The patient was then intubated and ventilated. His family reported an additional history of acupuncture for back pain, which sustained an inflamed wound on his right forearm. Abdominal computed tomography was performed, which confirmed bilateral iliopsoas abscess without involvement of intra-abdominal organs. A preliminary report of blood culture revealed Gram-positive cocci. Echocardiography showed vegetation on the aortic valve, and moderate aortic regurgitation was sustained. He was started on vancomycin along with piperacillin–tazobactam. Ultrasound-guided percutaneous drainage was inserted into the bilateral abscess. Pus and blood yielded methicillin-sensitive *Staphylococcus aureus*. He remained septic. The repeat computed tomography showed the right abscess enlarged. A repeated echocardiogram showed that the vegetation increased. Further incision and surgical drainage were performed with continuous wash-out.

**Outcome:**

His condition improved after management and he was discharged to a regional hospital for ongoing care.

**Conclusion:**

Prompt diagnosis and surgical treatment are essential to improve patient outcomes. The unique aspect of this case is the persistence of the methicillin-sensitive *Staphylococcus aureus* infection. Centralized surgical services are pivotal in conjunction with robust antimicrobial regimens.

**Lesson:**

This case reinforces the importance of high clinical suspicion of an unknown source of sepsis.

## 1. Introduction

An iliopsoas abscess (IPA) is an uncommon but lethal medical condition. Infection in the psoas muscle is associated with its unique anatomy. Pathogens can access the muscle directly or via hematogenous seeding. The psoas muscles arise from the lateral borders of the 12th thoracic to the 5th lumbar vertebrae and extend through the pelvic retroperitoneum and over the pelvic brim to be inserted into the lesser trochanter of the femur. Therefore, it is adjacent to multiple retro-abdominal and intra-abdominal organs, including the kidneys, ureters, pancreas, appendix, and bowel. Infections in these organs tend to extend directly into the psoas muscle. Furthermore, hematogenous spread from distant organs predisposes to an abundant blood supply to the muscle.^[1]^ The clinical presentation of IPA is variable and nonspecific. The typical clinical triad consists of fever, back pain, and limping, which only occurs in 30% of cases of IPA.^[1]^ Because of innervation of the 2nd to 4th lumbar vertebrae, the pain can radiate to the groin, and vague abdominal pain can be present. Treatment includes surgical or percutaneous drainage and prompt administration of antibiotics. IPA can be complicated by sepsis and multiorgan failure, which is related to delayed diagnosis and multiple comorbidities. The mortality rate was reported as 5% to 15% and relapse rate as15.8%, respectively.^[2]^ IPA is classified as primary or secondary, depending on the presence of infection in an adjacent organ. Previous studies have revealed a high prevalence of primary IPA in Asia.^[2,3]^ *Staphylococcus aureus* is the most common pathogen causing primary IPA, which is prone to cause septic shock and has a higher mortality rate than other pathogens.^[2]^ This case demonstrates the fulminate clinical course of *S aureus* infection in the iliopsoas muscle. Centralized surgical services are highlighted in treatment. Furthermore, the paucity of recommendations and controversy regarding appropriate antimicrobial therapy are notable features of this case.

## 2. Ethics approval and consent to participate

Informed written consent was obtained from the patient and the next of kin for the publication of this case report and accompanying images. The study was reviewed and approved by the local ethics committee of the Guangdong Provincial People’s Hospital Zhuhai Hospital (Jinwan Central Hospital of Zhuhai). All procedures performed in studies involving human participants were in accordance with the ethical standards of the Guangdong Provincial People’s Hospital, Zhuhai Hospital (Jinwan Central Hospital of Zhuhai) and/or national research committee and with the 1964 Helsinki Declaration and its later amendments or comparable ethical standards.

## 3. Case presentation

A man aged 65 years, resident in Zhuhai presented to the emergency department with a 2-day history of significant lower back pain, numbness in the left leg, and decreased ambulation. His medical history was unremarkable. He was afebrile and his vital signs were stable. Upper limb examination confirmed normal power, tone, and reflexes with an intact sensation. Lower limb examinations showed reduced power of the left hip flexors (2/5) and reduced sensation in the left L2 to L4. On palpation, tenderness was found at the 4th to 5th lumbar vertebrae and bilateral sacroiliac joints, with a limited range of motion at the left hip and knee. Rectal examination revealed normal anal tone and sensation. He was admitted to the orthopedics department with a provisional diagnosis of lumbar disc herniation with sciatica in December 2023. Analgesics were administered regularly.

However, his condition deteriorated on the 5th day of hospitalization. He reported significant shortness of breath and desiderated 76% on a Hudson mask at a temperature of 39 °C. Piperacillin–tazobactam was empirically administered after the initial screening for sepsis. However, his hypoxemia did not improve. The patient became delirious with a Glasgow comatose score (GCS) of 10 and tachycardiac, with an ongoing fever. He was transferred to the intensive care unit (ICU) for intubation and ventilation on the 5th day of hospitalization. The patient received fluid resuscitation, and inotropic support was initiated.

**Figure 1.**
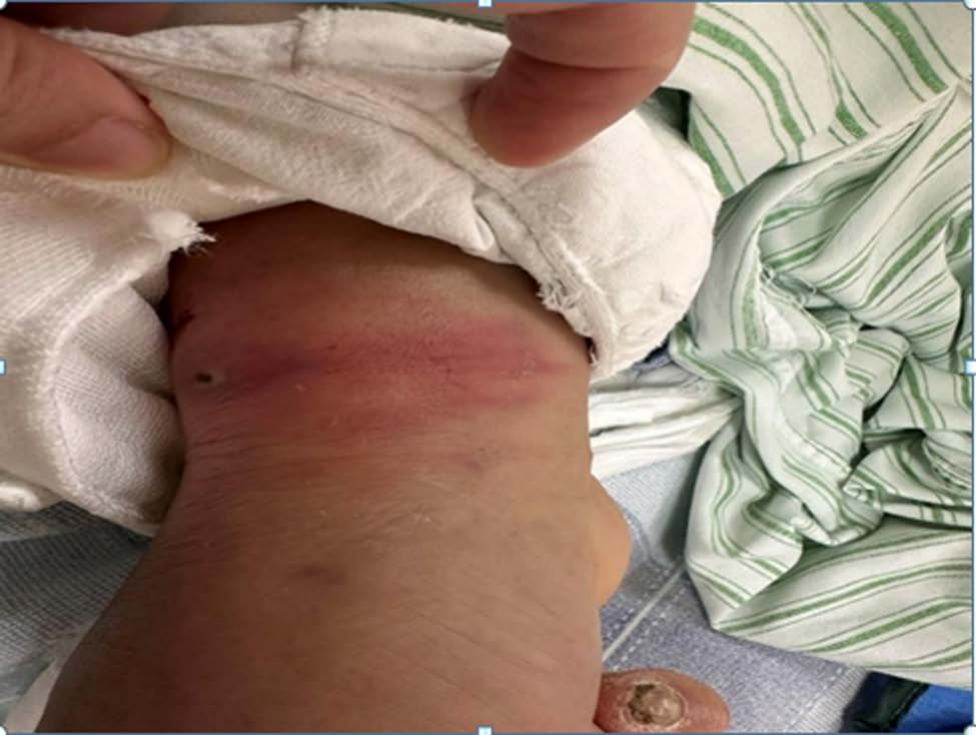
Cellulitis on right forearm.

**Figure 2.**
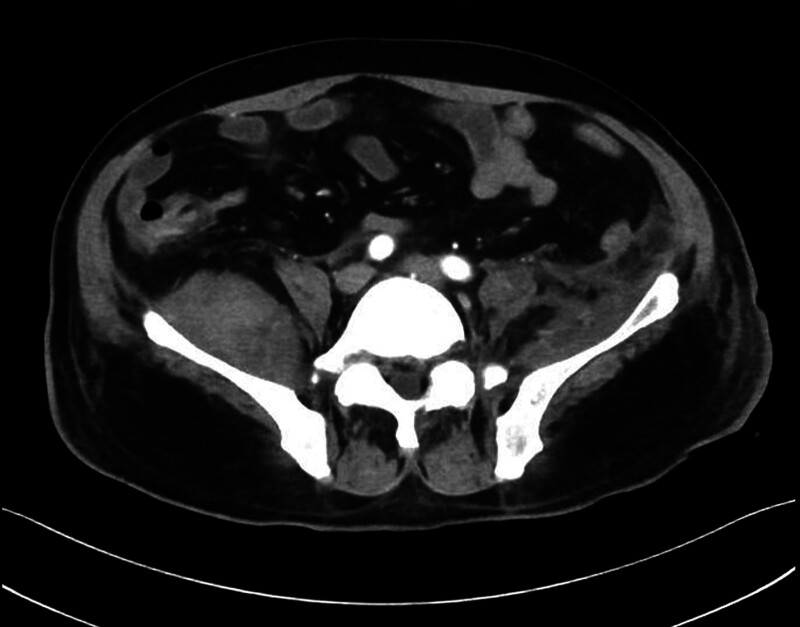
CT scan showed iliopsoas abscess. CT = computed tomography.

**Figure 3.**
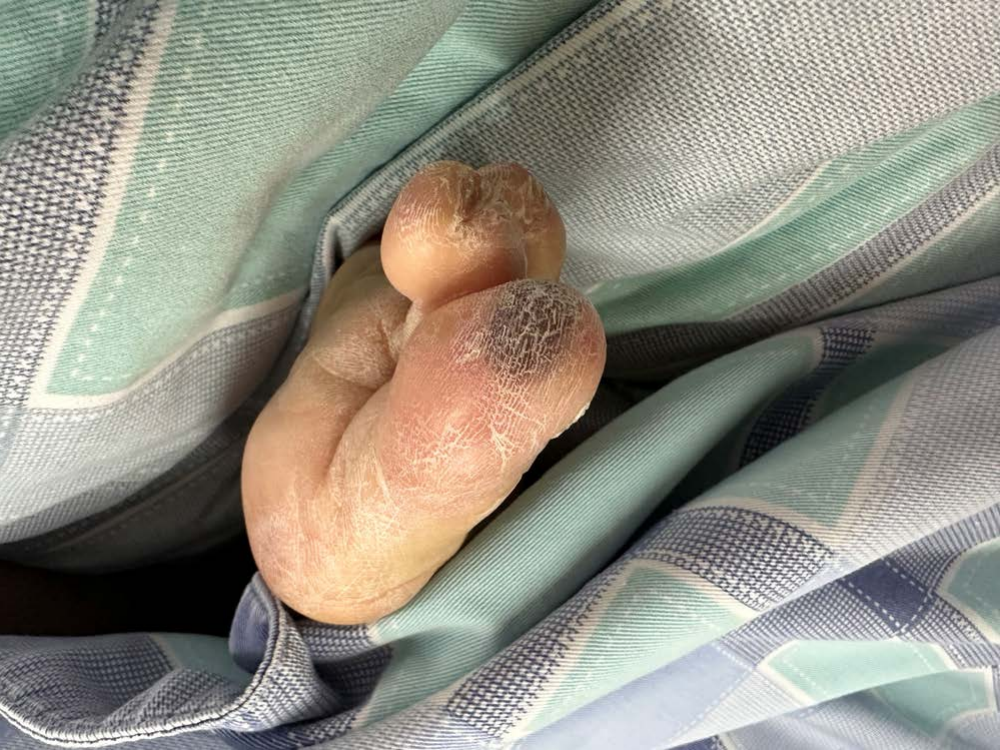
Janeway lesion was found on the patient’s right toe.

Upon admission to the ICU, his family reported an additional history of acupuncture in a clinic for back pain before his presentation. Upon examination, cellulitis was found on his right forearm, which appeared erythematous at a size of 3 × 2 cm with a central necrotic eschar (Fig. 1). Hematological investigations confirmed an elevated C-reactive protein (CRP) at 281 mg/L and acute kidney injury. An arterial blood gas showed: PH 7.39, base excess-6.3 mmol/L, HCO_3_ 19.6 mmol/L, lactate 2.1 mmol/L, PaO_2_ 60 mm Hg, PaCO_2_ 32 mm Hg. The HIV serological test results were negative. Meanwhile, the patient underwent a computed tomography (CT) scan, which showed bilateral avascular necrosis of the femoral head (stage IV) with subluxation of the right hip, abscess of the left iliac muscle, and left hip joint formation with extensive edema involving the pelvic presacral fat space, left pubis muscle, iliopsoas muscle, gluteus minimus, gluteus medius, obturator external muscle, adductor major muscle, adductor longus muscle, and adductor brevis muscle (Fig. 2). A Janeway lesion was found in the right toe (Fig. 3). Bedside echocardiography revealed moderate aortic valve regurgitation with a 5 × 7 mm vegetation on the right coronary valve. The anterior leaflet of the mitral valve and the subvalvular chordae tendinea appeared frizzy. Blood culture was collected on completion of echocardiography. Therefore, piperacillin/tazobactam was maintained for full coverage of pathology in the chest, muscle, and valve.

**Figure 4.**
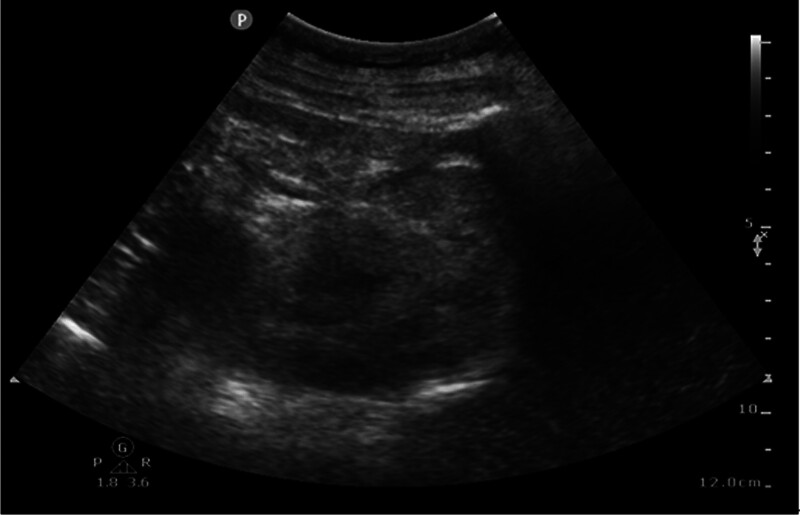
Bedside ultrasound showed an abscess of 7 × 8 cm.

**Figure 5.**
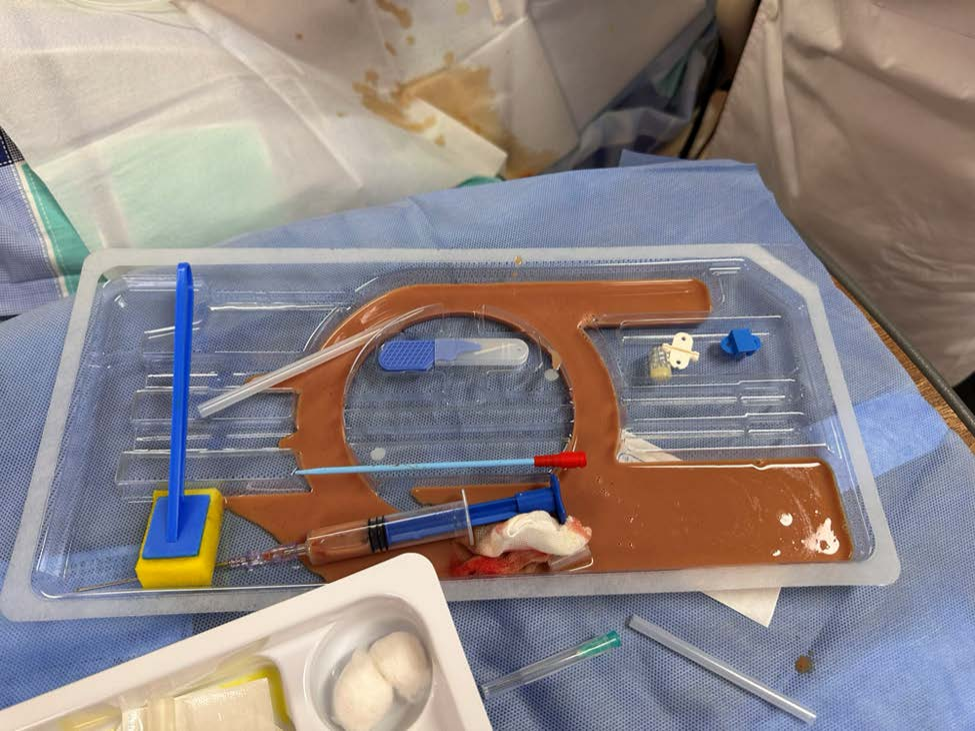
Coffee-ground pus was drained from the left side abscess.

On the 6th day of hospitalization, a preliminary blood smear revealed Gram-positive cocci. Given the history of acupuncture, methicillin-resistant *S aureus* (MRSA) infection was possible; therefore, vancomycin was added for bloodstream infection and IPA. After discussions with his family, his next of kin concurred with abscess drainage on the 7th day of hospitalization, and consent was finally obtained. Fine needle drainage under ultrasound guidance was performed on the left psoas abscess, which was 7 × 8 cm in size (Fig. 4). Approximately 200 mL of coffee-ground pus drained from the left psoas abscess (Fig. 5) over the 1st day after insertion. For the right side of IPA, ultrasound-guided drainage was inserted on the 8th day for the right IPA with a size of 6 × 7 cm but with a minimum drain. However, the patient remained septic and his inflammatory markers remained elevated with CRP at 228 mg/L and procalcitonin at 2.4 ng/mL.

**Figure 6.**
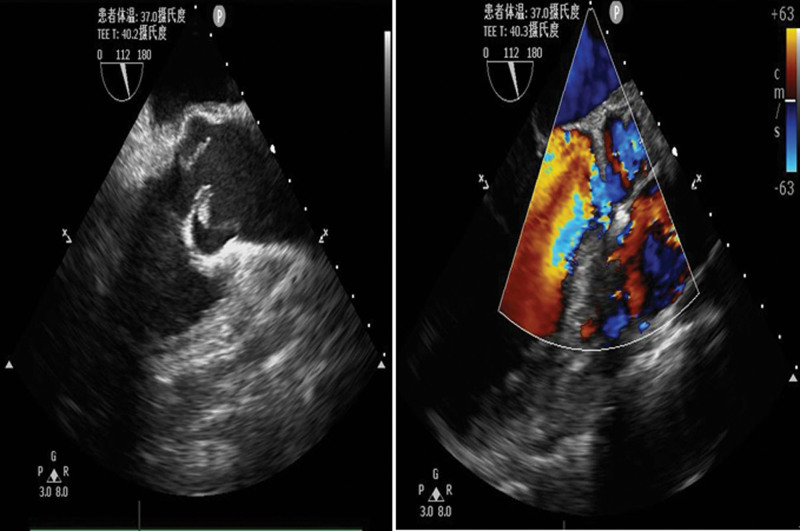
TEE showed significant aortic regurgitation and vegetation. TEE = transesophageal echocardiography.

On the 9th day, pus and blood cultures showed growth of *S aureus*, which was sensitive to methicillin, cefazolin, vancomycin, teicoplanin, and tigecycline. Contrast-enhanced abdominal CT showed that the abscess in the left psoas muscle, bilateral iliolumbar, and internal iliac muscles appeared more extensive, involving the bilateral retroperitoneal area, bilateral psoas major muscle, and bilateral iliac muscles. Above all, the infection remained significant and showed a poor response to the current antibiotic regimens. The drainage appeared thick and scant with multiple separations in the abscess in our bedside ultrasound scan. Therefore, the orthopedics team advised for open incision and drainage of the iliac fossa abscess. In the operation, debridement was performed on the abscess which appeared necrotic, purulent and extensive in iliopsoas muscles with multiple separations in the abscess. Continuous irrigation of the abscess was performed using the normal saline solution for 24 hours following the operation. On the 10th day, we repeated a bedside ultrasound, which showed the size of the bilateral iliac fossa abscess was reduced compared to the preoperative image. Transesophageal echocardiography (TEE) was performed to show that the aortic valve vegetation had progressed to a size of 9 × 6 mm with military nodules on the mitral valve leaflets (Fig. 6).

Given adequate antibiotics and drainage, the patient became afebrile as inflammatory markers and oxygenation improved. His inflammatory markers have been declining since the 11th day (2 days after the operation) with CRP at 123 mg/L and procalcitonin at 2.01 ng/mL. The drainage appeared serous postoperatively. His general condition improved after operation. His arterial blood gas profile improved significantly, with PH 7.48, base excess-0.8 mmol/L, HCO_3_ 23.5 mmol/L, lactate 0.7 mmol/L, PaO_2_ 138 mm Hg, PaCO_2_ 30 mm Hg. Bedside echocardiogram showed the size aortic valve vegetation was stable, for which the cardiothoracic team consulted and recommended observation and ongoing antibiotic administration. He was extubated on the 15th day of hospitalization. His condition improved and discharged to a regional hospital close to his family for ongoing care.

## 4. Discussion

An IPA, a collection of pus within the compartment of the psoas and iliacus muscles, was first described by Mynter et al.^[4]^ IPA is a rare medical condition with an incidence rate of 0.4 per 100,000 individuals reported in the United Kingdom. Delays in diagnosis are the major cause of morbidity and can be lethal.^[5]^ By reviewing previous literature, we found that making an early diagnosis of IPA has been challenging due to insidious onset and nonspecific clinical presentation. In general, IPA has a subacute clinical course, which results in delayed action for medical assistance in an average of 5 to 6 days after the onset of symptoms.^[5]^ Pyomyositis including IPA consists of 3 consecutive clinical stages. The onset is generally insidious with pain in the muscles involved. The classic symptoms of fever, flank pain, and limited hip movement, known as the psoas-muscle sign, are present in only 30% of patients, making diagnosis challenging.^[6]^ Unless diffuse muscle infection, few patients present in this stage. Most patients present in the 2nd stage when muscular symptoms are more prominent due to the formation of abscesses. Many patients often experience a gradual onset of nonspecific symptoms, such as malaise and low-grade fever, which may eventually develop into more specific manifestations such as abdominal discomfort, discomfort in the flank region, a flexed and externally rotated hip, and pain upon movement of the hip. This pain can be caused by irritation of the abdominal muscle and referred pain from nerve roots L2, L3, and L4, which innervate the psoas muscle. The thorough physical examination could alert physicians to consider pathology in the iliopsoas muscles at this stage. Given the deep anatomical location of the iliacus and psoas muscle bellies, their sheaths, and conjoined tendons, examination can be challenging. However, abdominal tenderness, antalgic passive hip movements, and occasionally a painless subinguinal mass can be elicited.^[7]^ The 3rd stage is characterized of systemic toxic symptoms including high fever or even septic shock, whereas patients generally deteriorate rapidly at this stage, leading to the high fatality rate. We failed to diagnose our patient with IPA on admission as some important points in medical history and physical examination were missed. The patient presented with atypical back pain without fever and was initially misdiagnosed with sciatica. The history of acupuncture and physical examination findings of his forearm could have been a clue that facilitated an early diagnosis of infection. A lesson should be learned that an accurate history and thorough physical examination are crucial for raising the suspicion of an IPA.

IPA can be categorized as primary or secondary. Our patient was diagnosed with a primary IPA. It is pivotal to identify the category of IPA once diagnosed, as it assists in deciding on an antibiotic regime. Primary IPA occurs when a causative organism spreads hematogenously or lymphatically from a remote site. In contrast, secondary IPA arises because of the direct extension of a nearby infectious or inflammatory process into the iliopsoas muscle.^[8,9]^ Secondary IPA constitutes the majority of cases and typically results from intra-abdominal inflammatory processes, particularly those originating from the intestines. Other causes include spinal and skeletal pathologies.^[4,10,11]^ An overview of the conditions associated with the development of secondary IPA is presented in Table 1.^[13]^

**Table 1:** Table indicating documented origins for secondary spread to the iliacus or psoas muscles.^[12]^

Source location	Condition
Gastrointestinal	Crohn disease
	Diverticulitis
	Appendicitis
	Colorectal carcinoma
Genitourinary	UTI
	Instrumentation
Musculoskeletal infection	Vertebral osteomyelitis
	Infectious sacroilitis
	Septic arthritis
Others	Trauma
	Endocarditis
	Hepatocellular carcinoma
	Femoral artery catheterization

The management of primary IPA should consist of the early use of appropriate antibiotics along with abscess drainage. Previous literature elaborated on the microbiology findings according to the origin of the abscess which was depicted in Table 2.^[13]^ The most frequently encountered organism in both primary and secondary IPA (with skeletal infection as the source) is *S aureus*.^[7,10,14]^ Among the 2 most common etiologies of secondary IPA, specifically gastrointestinal and urinary IPA, *Escherichia coli* is the most prevalent organism.^[7,10,14]^ Other organisms that have been observed include Bacteroides, *Mycobacterium tuberculosis*, *Enterococcus faecalis*, *Peptostreptococcus*, and *Streptococcus viridans*.^[7,10,14,15]^ The most common organism of primary IPA is *S aureus* and the literature recommends the administration of broad-spectrum antibiotics that are effective against potential microorganisms, particularly on *S aureus*.^[13]^ Our patient presented with significant systemic toxic symptoms with the manifestation of endocarditis and this is consistent with significant *S aureus* infection according to previous literature. The patient received vancomycin as anti-staphylococcus regime, while the literature recommends cefazolin for IPA with *S aureus*. The rationales for our antimicrobial regime included risk factors of patient and local microbial epidemiology. First of all, our case highlighted the history of acupuncture therapy and the patient might be predisposed to hematogenous seeding with *S aureus*. In addition, nosocomial MRSA infections in the local hospitals have been trending up over the last decade. Similar epidemic findings were reported in the previous literature. The most recent study by Lee et al,^[16]^ which observed 126 patients diagnosed with IPA, showed that the microbiological corroboration rate in IPA patients was 52.8% with positive blood culture and 78.3% with positive pus culture. The causative organisms were predominantly Gram-positive bacteria, with *S aureus*, because more than 60% of these cases were mostly isolated from primary and skeletal origin. In *S aureus* cases, MRSA accounted for 26.8% of blood cultures and 35% of abscess aspirate cultures; both prevalence rates were comparable to those of the general population.^[17–19]^ Although some studies have suggested that empiric coverage of MRSA is not warranted for all IPA patients because of the low frequency,^[12,14]^ other recent articles have indicated a significant increase in MRSA isolated from IPA.^[18,19]^ This may be due to several reasons, such as drug-resistant organisms, repeat surgery, and an increasing number of immunocompromised hosts.^[19–21]^ In light of local epidemiology and patient’s condition, vancomycin was administered for MRSA in this case.

**Table 2:** Microbiology and outcome of iliopsoas abscess in 124 patients shows the 7 most common organisms, their origins, and proportion of that origin.^[12]^

Microorganism	Primary abscesses (%)	Skeletal origin (%)	Gastrointestinal origin (%)	Urinary origin (%)
*Staphylococcus aureus*	42.9	35.2	–	7.7
*Escherichia coli*	14.3	2.9	42.1	61.5
*Bacteroides* spp	4.8	–	26.3	15.4
*Mycobacterium tuberculosis*	4.8	17.7	–	–
*Streptococcus viridans*	19	–	10.5	–
*Enterococcus faecalis*	–	–	15.8	15.4
*Peptostreptococcus* spp	4.8	2.9	15.8	–

In terms of drainage, no standard protocol has been established for primary IPAs. Treatments often differ due to differences in the abscess size, pathogen type, invasion extent, and infection severity. Historically, surgical drainage was the preferred treatment method, and some authors have reported faster recovery rates after open drainage procedures compared to antibiotics alone.^[16]^ However, image-guided percutaneous drainage has been demonstrated to be a highly effective and safe alternative.^[22]^ Nevertheless, there are instances where open drainage is preferable, particularly where IPA is a complication of an intra-abdominal disease process that also necessitates surgical intervention, such as complex Crohn disease or diverticulitis.^[23,24]^ There are also technical constraints associated with CT-guided drainage, such as dealing with small abscesses, multiple separations within the abscess, or inaccessible abscess locations.^[25,26]^ Overall, the current literature suggests that many small abscesses can be effectively managed with antibiotics alone, and for abscesses that require drainage, the majority can be aspirated effectively under CT guidance.^[14]^

In our case, surgical intervention was not performed in the first place because of the hemodynamic compromise. Some experts suggest that targeted antibiotics may suffice in treating abscesses measuring up to 60 mm^[27]^; however, without aspirating the abscess, the choice of antibiotics often remains a general estimation rather than a precise targeted therapy. Our patient had dissemination of the methicillin-sensitive *S* aureus (MSSA) infection presented as an increase in the size of the abscess, and endocarditis was observed even though targeted antibiotics were administered. Therefore, surgical intervention is indicated in conjunction with an antimicrobial system. Priority was given to ultrasound-guided percutaneous drainage in our patient due to minimal invasiveness and better tolerance in critically ill patients; however, the drainage was unsatisfactory due to the thickness of the pus. Therefore, surgical drainage was performed as a rescue procedure. With sufficient drainage of the abscess, clinical improvement was observed following the operation and this could justify the importance of an individualized strategy for drainage.

A few limitations in our study should not be overlooked. First, it is a retrospective case report, of which the outcome may not be reflective of the situation of the entire community. Clinical decisions on antibiotics and drainage should be made on a case-by-case basis. Second, to optimize antimicrobial therapy, the virulence factor test of *S aureus* should have been performed, which might assist in understanding the high virulence of MSSA infection. However, the test was not conducted due to limited laboratory conditions.

## 5. Conclusion

IPA is a medical emergency requiring early recognition for the initiation of timely treatment and reduction of associated morbidity and mortality. The antimicrobial regimen should be based on the etiology of the abscess. The initial treatment for IPA typically involves the administration of broad-spectrum antibiotics that are effective against potential microorganisms, particularly *S aureus*. Prompt surgical drainage combined with antibiotic therapy is crucial to resolve infection and prevent potential complications.

## Author contributions

**Writing – original draft:** Wenyu He.

**Writing – review & editing:** Wenyu He, Ye Yuan.

**Resources:** Jihua Huang.
